# Lumen-apposing metal stent for drainage of a perirectal abscess complicating endoscopic ultrasound-guided fine-needle biopsy

**DOI:** 10.1055/a-2686-3145

**Published:** 2025-09-09

**Authors:** Marcin Polkowski, Mateusz Szmit, Krzysztof Skoczylas, Jakub Krzyżkowiak, Jakub Pałucki, Jarosław Reguła

**Affiliations:** 1 Department of Gastroenterology, Hepatology and Clinical Oncology, Center of Postgraduate Medical Education, Warsaw, Poland; 2 Department of Oncological Gastroenterology, The Maria Skłodowska-Curie National Research Institute of Oncology, Warsaw, Poland; 3 Department of Radiology, The Maria Skłodowska-Curie National Research Institute of Oncology, Warsaw, Poland

Abscess formation is a rare complication following lower gastrointestinal tract endoscopic ultrasound (EUS)-guided tissue acquisition 1 . Transrectal EUS-guided drainage has been shown to be a safe and effective treatment for postsurgical and inflammatory pelvic fluid collections 2 3 . We report a case of an abscess complicating fine-needle biopsy (FNB) of a perirectal mass, successfully managed with EUS-guided transrectal drainage using a lumen-apposing metal stent (LAMS).

A 69-year-old man with a history of prostatectomy and radiotherapy for prostate cancer 4 years earlier, and proctosigmoidectomy with colostomy for rectosigmoid cancer 2 years earlier, underwent transrectal EUS-FNB of a perirectal mass suspected to represent a cancer recurrence. Two needle passes through the rectal stump yielded necrotic material without malignant features. Several days later, the patient presented with severe perirectal pain, leukocytosis, and elevated C-reactive protein. Computed tomography (CT) revealed a perirectal abscess, 50 mm × 45 mm in size, at the site of previously biopsied necrotic lesion ( Fig. 1 ). Due to its proximity to the rectal stump, transrectal EUS-guided drainage was performed ( Video 1 , Fig. 2 ). The abscess was punctured for culture using a 19-G FNA needle (EZ Shot 3 plus, Olympus), followed by placement of a 20 mm × 10 mm cautery-enhanced LAMS using a freehand technique (Hot Axios, Boston Scientific). The abscess cavity was irrigated with saline, and a 7-Fr, 5-cm double-pigtail plastic stent was placed coaxially through the LAMS. The patient experienced immediate pain relief, and his post-procedure course was uneventful. A follow-up CT scan two weeks later confirmed resolution of the abscess ( Fig. 3 ). Endoscopic examination through the LAMS revealed a collapsed, residual cavity lined with granulomatous tissue ( Fig. 4 ). Both stents were removed using rat-tooth forceps. The resulting wall defect was left to heal spontaneously ( Fig. 5 ). At 6-week follow-up, clinical and magnetic resonance imaging assessment confirmed full recovery.

**Fig. 1:**
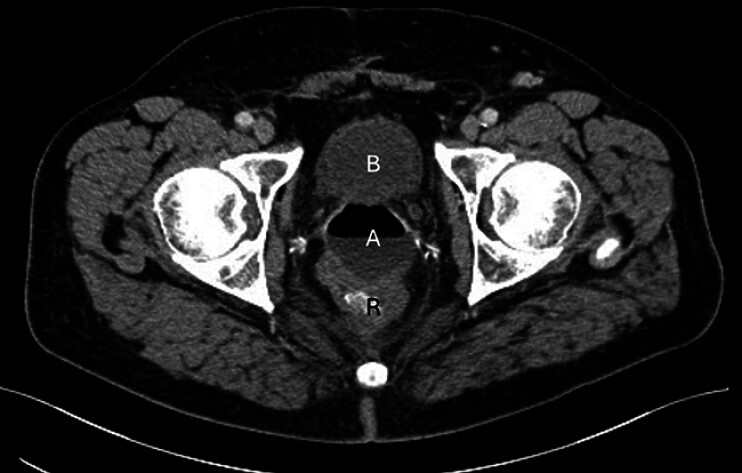
Computed tomography image of a perirectal abscess (A) with an air-fluid level, located between the urinary bladder (B) and rectal stump (R), at the site of the previously biopsied perirectal necrotic lesion.

Transrectal drainage of a perirectal abscess using endoscopic ultrasound-guided lumen-apposing metal stent.Video 1

**Fig. 2:**
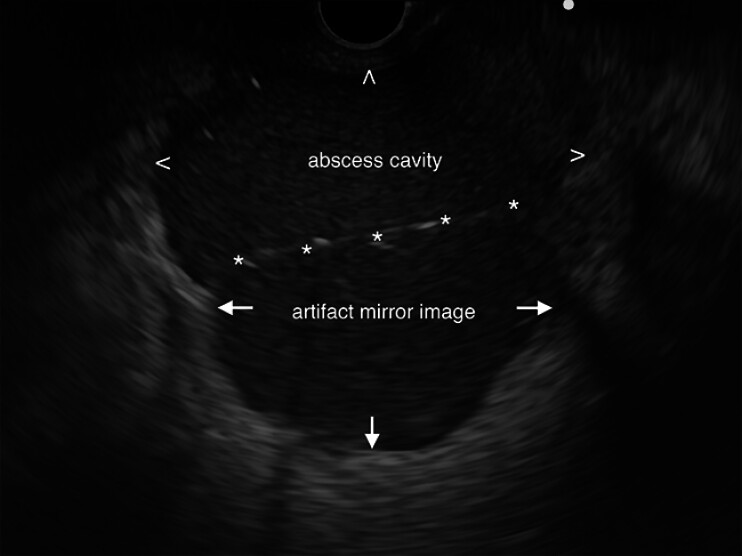
Endoscopic ultrasound image of the abscess (arrowheads), with mirror-image artifact (arrows) arising at the highly reflective interface between gas and fluid in the abscess cavity (asterisks).

**Fig. 3:**
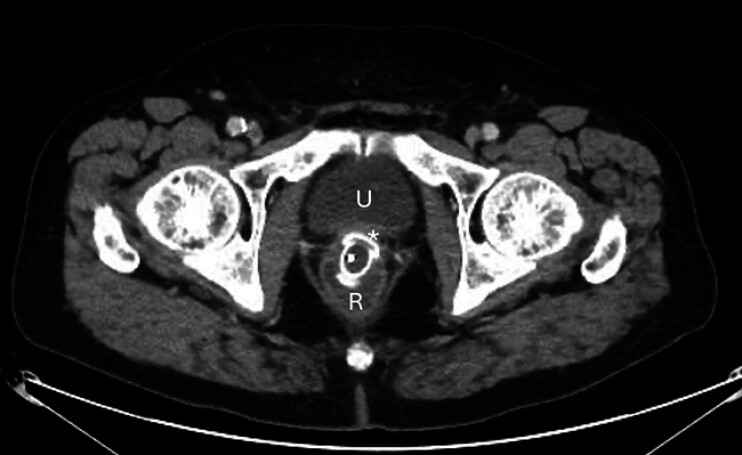
Computed tomography image two weeks after drainage, showing the lumen apposing metal stent connecting the residual abscess cavity (asterisk) with the rectal stump (R). “U” denotes the urinary bladder.

**Fig. 4:**
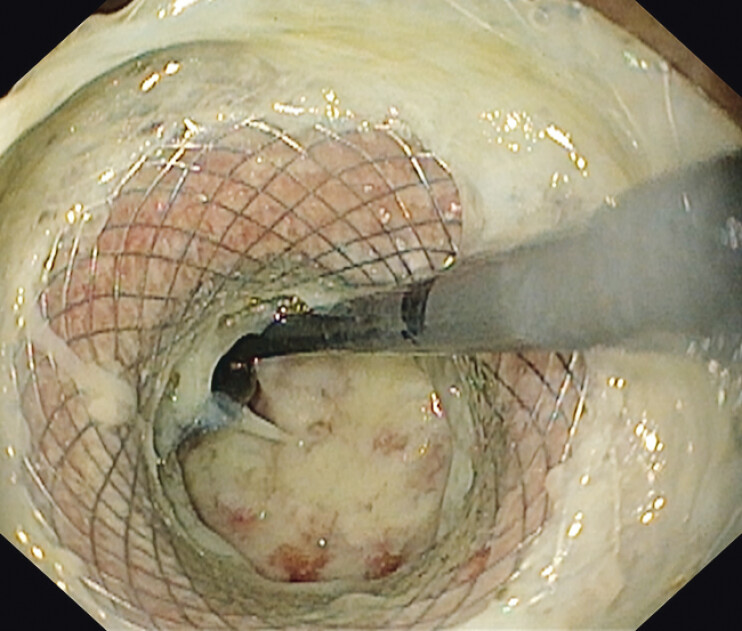
Endoscopic image of the rectal stump 14 days after drainage. The residual abscess cavity lined with granulomatous tissue is seen through the lumen-apposing metal stent.

**Fig. 5:**
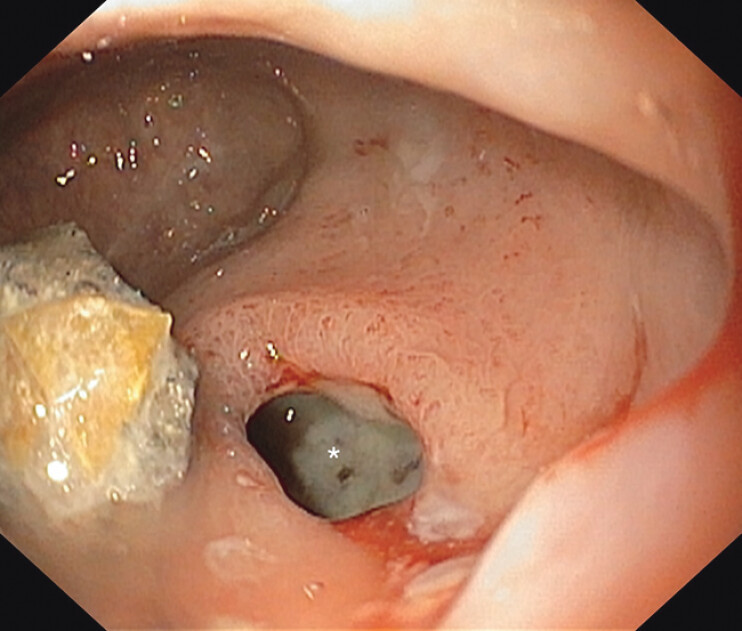
Endoscopic image of the rectal stump immediately after removal of the lumen-apposing metal stent. The resulting wall defect (asterisk) was left to heal spontaneously.

Endoscopy_UCTN_Code_TTT_1AS_2AZ

## Notes

Endoscopy E-Videos https://eref.thieme.de/e-videos
*E-Videos* is an open access online section of the journal *Endoscopy* , reporting on interesting cases and new techniques in gastroenterological endoscopy. All papers include a high-quality video and are published with a Creative Commons CC-BY license. Endoscopy E-Videos qualify for HINARI discounts and waivers and eligibility is automatically checked during the submission process. We grant 100% waivers to articles whose corresponding authors are based in Group A countries and 50% waivers to those who are based in Group B countries as classified by Research4Life (see: https://www.research4life.org/access/eligibility/ ). This section has its own submission website at https://mc.manuscriptcentral.com/e-videos .
